# Pleural cancer mortality in Spain: time-trends and updating of predictions up to 2020

**DOI:** 10.1186/1471-2407-13-528

**Published:** 2013-11-06

**Authors:** Gonzalo López-Abente, Montserrat García-Gómez, Alfredo Menéndez-Navarro, Pablo Fernández-Navarro, Rebeca Ramis, Javier García-Pérez, Marta Cervantes, Eva Ferreras, María Jiménez-Muñoz, Roberto Pastor-Barriuso

**Affiliations:** 1 Environmental and Cancer Epidemiology Unit, National Centre for Epidemiology, Carlos III Institute of Health, Monforte de Lemos 5 28029, Madrid, Spain; 2 Consortium for Biomedical Research in Epidemiology and Public Health (CIBER en Epidemiología y Salud Pública - CIBERESP), Madrid, Spain; 3 Ministry of Health, Social Services and Equality, Paseo del Prado 18-20, Madrid 28014, Spain; 4 Departament of History of Science, Granada University, Avda. de Madrid 11, Granada 18012, Spain; 5 Division of Health Research, Faculty of Health and Medicine, Lancaster University, Lancaster LA1 4YB, UK

**Keywords:** Age-period-cohort, Asbestos, Epidemiology, Pleural cancer, Mesothelioma

## Abstract

**Background:**

A total of 2,514,346 metric tons (Mt) of asbestos were imported into Spain from 1906 until the ban on asbestos in 2002. Our objective was to study pleural cancer mortality trends as an indicator of mesothelioma mortality and update mortality predictions for the periods 2011–2015 and 2016–2020 in Spain.

**Methods:**

Log-linear Poisson models were fitted to study the effect of age, period of death and birth cohort (APC) on mortality trends. Change points in cohort- and period-effect curvatures were assessed using segmented regression. Fractional power-link APC models were used to predict mortality until 2020. In addition, an alternative model based on national asbestos consumption figures was also used to perform long-term predictions.

**Results:**

Pleural cancer deaths increased across the study period, rising from 491 in 1976–1980 to 1,249 in 2006–2010. Predictions for the five-year period 2016–2020 indicated a total of 1,319 pleural cancer deaths (264 deaths/year). Forecasts up to 2020 indicated that this increase would continue, though the age-adjusted rates showed a levelling-off in male mortality from 2001 to 2005, corresponding to the lower risk in post-1960 generations. Among women, rates were lower and the mortality trend was also different, indicating that occupational exposure was possibly the single factor having most influence on pleural cancer mortality.

**Conclusion:**

The cancer mortality-related consequences of human exposure to asbestos are set to persist and remain in evidence until the last surviving members of the exposed cohorts have disappeared. It can thus be assumed that occupationally-related deaths due to pleural mesothelioma will continue to occur in Spain until at least 2040.

## Background

Pleural cancer mortality is an acknowledged indicator of exposure to asbestos and mesothelioma mortality [1], and since the 1960s mesothelioma has been gaining interest world-wide as a result of its increasing incidence, related medico-legal issues and poor prognosis [2]. Pleural cancer can rapidly prove fatal, as it has five- and ten-year relative survival rates of 6.8% and 2.5% respectively [3]. Most mesotheliomas are due to exposure to asbestos, with 80%-85% of cases being attributable to occupational exposure [4]. Increased risk has been reported for workers employed in asbestos mines, asbestos plants, the installation and manufacture of insulation materials, the production of anti-gas masks, shipyards, railways and other occupations involving inhalation of asbestos dust [4-6].

From 1906 until 2002, the year in which asbestos was banned, a total of 2,514,391 Mt of asbestos were imported into Spain, with a halt in imports during the years of the Spanish Civil War (1936–1939). In view of the country’s lack of domestic production, import data are the best indicator of asbestos consumption in Spain. The vast majority of the imported asbestos was in raw form (fibre, dust) and was earmarked for manufacturing products, ranging from cement, insulation and textiles to ships and automobiles. Imports increased steadily from 1906 until 1974 and peaked in the five-year period 1973–1977, with an average of 113,921 tons per year and a maximum of 130,293 tons in 1974 [7]. This figure was slightly lower than that recorded in 1975 for neighbouring countries, such as Italy (132,184 Mt), France (136,587 Mt) and the United Kingdom (137,487 Mt) [8]. In Spain, as in Italy and France, imports decreased gradually from 1980 onwards, while in other countries such as the USA, Australia, United Kingdom and Scandinavia, the decline began some 10 to 20 years earlier [9].

In terms of the number of workers exposed in Spain, in 1971 initial estimates put the number of exposed subjects at 8,000, 70% in the fibre-cement sector [10]. In 1991, the National Institute for Health & Safety in the Workplace (*Instituto Nacional de Seguridad e Higiene en el Trabajo*) estimated the number of exposed workers at 60,488 [11], while the CAREX (CARcinogen EXPosure) project estimated this figure as being 56,600 by the end of 1990s [12]. Moreover, in December 2008 the Comprehensive National Health Surveillance Programme of Workers exposed to asbestos in Spain (*Programa Integral de Vigilancia de Salud de Trabajadores Expuestos a Amianto en España*/*PIVISTEA*) included a total of 22,158 workers from 14 Autonomous Regions (*Comunidades Autónomas*) and 306 companies [13].

Neighbouring countries have drawn up mesothelioma mortality predictions, which are periodically updated [2,9,14,15]. These predictions are based on age-period-cohort analyses of pleural cancer mortality and the examination of its association with asbestos consumption [9,16]. The updating of predictions is a result of the extreme sensitivity of the models to the introduction of the most recent data. A pleural cancer mortality forecast was recently published (2008) in Spain covering the period 2002–2016 [17].

Accordingly, the aim of this study was to analyse pleural cancer mortality for the purpose of: 1) analysing changes in period and cohort effects on pleural cancer mortality; and, 2) updating predictions of pleural cancer and pleural mesothelioma mortality for the five-year periods 2011–2015 and 2016–2020, using the age-period-cohort approach and national asbestos imports as an indicator of exposure.

## Methods

### Mortality and population data

Mortality data were drawn from the records of the National Statistics Institute (*Instituto Nacional de Estadística*/*INE*) for the study period (1976–2010), and corresponded to deaths coded as malignant pleural neoplasm, namely, codes 163 (International Classification of Diseases-9th Revision/ICD-9) and C38.4, C45.0 (ICD-10). Midyear populations and projections for the periods 2011–2015 and 2016–2020 were likewise obtained from the National Statistics Institute.

### Age-period-cohort (APC) models

Log-linear Poisson models were fitted to study the effect of age, period of death and birth cohort. Five-year age groups (35–39 to 85+ years) and periods were used (1976–80, 1981–85,…, 2006–10). To address the “non-identifiability” problem (the three factors, i.e., age, period and cohort, are linearly dependent), we used the evaluation of estimable parameters proposed by Holford [18], such as the curvature in each effect and the sum of period and cohort linear slopes, also known as net drift. Age groups <35 years were excluded from this analysis, due to the limited number of deaths in these age groups. We checked for extra-Poisson dispersion [19], and effects were calculated using a negative binomial distribution. Specific functions for all these analyses were written in R [20].

### Curvature change points

The presence of points of change in the curvatures of the cohort and period effects was evaluated using segmented regression. Details of the recursive algorithm used to estimate the segmented regression have been published elsewhere, and the procedure can be easily applied using the R package “segmented” [21]. These models were fitted to the relationship between curvature effects and time, thereby enabling the change points to be identified and their confidence intervals calculated.

### Age-period-cohort prediction model

**Figure 1:**
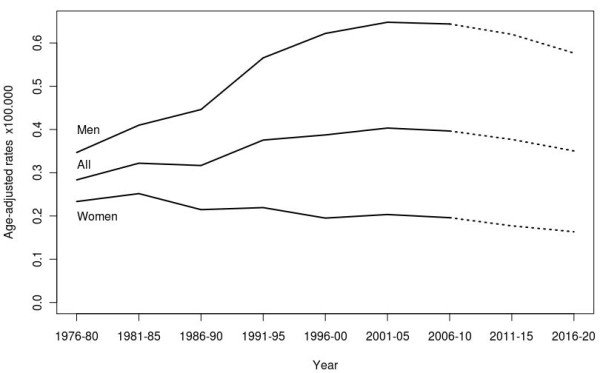
Age-adjusted pleural cancer mortality rates (1976–2010) and predictions (2011–2020) in Spain.

Prediction age-period-cohort models with 1/5-power link and Poisson variance [22] were fitted to the observed pleural cancer death rates by 5-year age groups (35–39 to 85+ years) and seven calendar periods (1976–1980 to 2006–2010). Instead of the usual log link, the fractional-power link was used to level off the exponential growth in rates over time resulting from the standard log-linear model. The number of deaths from pleural cancer in each 5-year age group for the upcoming five-year periods 2011–2015 and 2016–2020 was predicted from the above model, by putting curvatures for future periods and cohorts equal to the last curvatures estimated from the model, and projecting the drift over time. Since the increasing drift attenuated gradually over the observed study period (Figure 1), we projected the linear slope between the two most recent 5-year periods, rather than the overall net drift, to emphasise recent trends. In addition, this linear slope was reduced by 25% in projecting the period 2016–2020, to account for an eventual deceleration in long-term trends. The lower age limit was chosen so that there would be cases in all periods. For age groups below this limit, future rates were estimated using the observed average rates for the last two periods [22]. All these predictions were performed for men and women, jointly and separately, using the Nordpred R statistical software package [22].

### Asbestos-consumption-based prediction model

Since most pleural cancers are mesotheliomas specifically linked to occupational asbestos exposure, a model based on national asbestos consumption figures was also used to perform long-term predictions of the annual number of deaths from pleural cancer among Spanish men over the period 2012–2038. In view of the negligible domestic production and export of asbestos in Spain, annual per capita asbestos consumption was determined as annual imports of raw asbestos in any form from 1906 to 2002 divided by the corresponding midyear Spanish population. Data on asbestos imports in Spain were obtained from several sources: primarily from a compilation made by the Catalonian Regional Labour Authority and from a report entitled, “*Foreign trade statistics* for *Spain*”, which compiled annual asbestos import and export figures; and also from company records [7,23].

A locally-weighted, running-line smoother with 10% bandwidth (proportion of points in the plot which influence the smoother at each value) was applied to this asbestos consumption series, in order to smooth out short-term fluctuations and highlight long-term consumption trends [24]. To develop the prediction model, the annual number of male pleural cancer deaths from 1975 to 2011 was regressed (ordinary least squares regression analysis) on the smoothed annual per-capita asbestos consumption using different exposure lag periods from 30 to 40 years in 1-year increments. Different functional relationships were also used, including linear and quadratic terms for the untransformed and log-transformed exposure, identity and log links for the mean response, and additive normally distributed errors. From among the different exposure lags and functional relationships, we selected the prediction model with the lowest Akaike Information Criterion (AIC). Note that the likelihood functions, and hence the AIC statistics, were comparable across all fitted models, since the mean response was transformed through the log link function, rather than log-transforming the response variable itself [25]. The model chosen was used to predict the annual number of male pleural cancer deaths across the upcoming period 2012–2038, based on the preceding national asbestos consumption figures [9].

## Results

From 1975 to 2010, there were 6,037 pleural cancer deaths in Spain, with the breakdown showing 3,986 (66%) in men and 2,051 (34.0%) in women. The male/female ratio rose from 1.1 in 1975 to 2.5 in 2010.

Observed and predicted (APC model) pleural cancer deaths in both sexes by age group and period of death are shown in Table 1. Predicted deaths for the period 2011–2020 ranged from 257 to 265 deaths per year (187–193 among men). Predictions for 2016–2020 indicate a total of 1319 pleural cancer deaths. Figure 1 depicts the trend in age-adjusted rates and predictions (dashed line) (both sexes; men; and women): the age-adjusted rates showed a levelling-off from the period 2001–2005 among men, and a gradual decline from the 1980s onwards among women. Predictions for the periods 2011–2015 and 2016–2020 maintain the general trend in age-adjusted rates in both men and women.

**Table 1:** Observed pleural cancer deaths by age-groups and period of death (1976–2010) and predicted (2011–2020) in Spain

**Age group**	**1976-80**	**1981-85**	**1986-90**	**1991-95**	**1996-00**	**2001-05**	**2006-10**	**2011-15+**	**2016-20+**
0-4	0	0	1	0	1	0	0	**0.0**	**0.0**
5-9	1	1	0	0	0	0	0	**0.0**	**0.0**
10-14	0	1	0	0	1	0	0	**0.0**	**0.0**
15-19	2	2	0	1	1	0	0	**0.0**	**0.0**
20-24	1	2	2	2	1	1	0	**0.4**	**0.4**
25-29	5	1	4	3	3	2	2	**1.6**	**1.3**
30-34	9	3	12	10	0	2	4	**2.8**	**2.2**
35-39	11	6	7	9	8	6	3	**3.1**	**2.8**
40-44	9	12	17	8	30	17	5	**5.7**	**5.8**
45-49	19	23	19	41	30	36	32	**15.1**	**11.8**
50-54	46	51	41	59	52	61	55	**58.9**	**30.9**
55-59	52	60	68	74	79	99	91	**100.1**	**88.3**
60-64	66	81	94	112	110	108	146	**127.8**	**143.7**
65-69	82	83	109	110	158	142	159	**183.7**	**164.8**
70-74	84	109	98	143	172	209	184	**201.6**	**238.8**
75-79	67	98	91	119	157	200	260	**219.5**	**231.9**
80-84	26	67	63	105	81	149	167	**205.6**	**193.5**
85+	11	21	33	58	73	95	141	**158.0**	**209.9**
Total deaths	491	621	659	854	957	1127	1249	**1283.9**	**1326.1**
Age-adjusted rates^§^ per 100,000	0.283	0.322	0.317	0.376	0.387	0.403	0.396	**0.377**	**0.350**
Men									
Deaths	265	346	407	557	676	796	897	**933.9**	**964.3**
Age-adjusted rates per 100,000	0.347	0.410	0.446	0.566	0.622	0.648	0.644	**0.620**	**0.577**
Women									
Deaths	226	275	252	297	281	331	352	**345.1**	**354.5**
Age-adjusted rates per 100,000	0.233	0.252	0.215	0.219	0.195	0.203	0.196	**0.177**	**0.163**
Men									
Predicted deaths from asbestos import model	291.7	338.5	425.8	508.7	670.1	824.9	902.4	892.8	787.9

§ Age-adjusted rates with european standard population.

+Predicted deaths and age-adjusted rates (in bold) obtained from the fractional-power link APC models.

**Figure 2:**
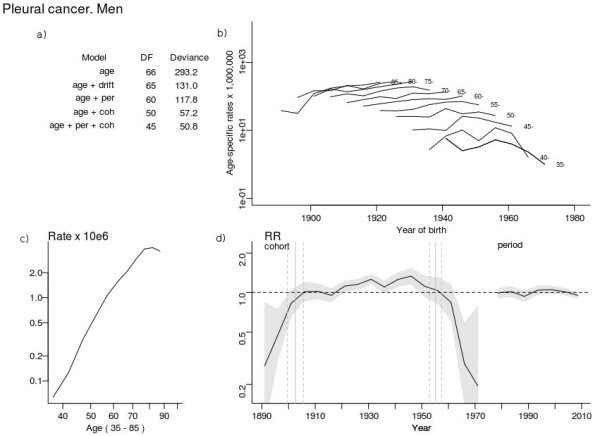
**Age-period-cohort analysis in pleural cancer mortality among men in Spain.** Deviance table for age-period-cohort models **(a)**, trends in age-specific rates by birth cohort **(b)**, cross-sectional age effect for an average period **(c)**, curvature and 95% confidence interval (grey shadow) of period and cohort effects **(d)**, and change points with 95% confidence intervals in cohort curvature (vertical grey lines).

The deviance changes in the sequential building of the models, the trend in age-specific death rates and the results for age, period, and cohort effects in men are all depicted in Figure 2a. Whereas the cohort-effect curvature was statistically significant (p < 0.001), this was not the case with the period-effect curvature (p = 0.28). Age-specific mortality rates showed an upward trend for persons aged over 50 years (Figure 2b) and a downward trend for the younger age groups, a rate pattern that corresponds to a cohort effect.

The graphical depiction of the cohort-effect curvature and its 95% confidence interval (Figure 2d) showed an increasing risk of death due to pleural cancer, rising to a maximum in the 1944–1948 cohorts and declining in subsequent generations. The drop was most pronounced from the 1956 cohort onwards. The segmented model detected two points of change in cohort-effect curvature (p < 0.001), at 1902.63 (95% Confidence Interval (CI) 1899.59-1905.66) and at 1955.21 (95% Confidence Interval (CI) 1953.01-1957.4) (grey lines in Figure 2d, with dashed lines showing the confidence interval). Points of change in the curvature of the period effect were not detected.

In women, the number of cases remained fairly stable over the study period and there was a slight decrease in age-adjusted rates. The number of estimated cases among men was 2.7 times higher than that among women. The shape of the curvature of the cohort effect (data not shown) in women was similar to that in men, with a point of change at 1956.47 (95% CI 1949.74-1963.19), and with no points of change in period-effect curvature.

The best statistical relationship between the annual per-capita asbestos imports (AI_*t*_) and the annual number of pleural cancer deaths (PCD_*t*_) among men was obtained from the model with a log link for the mean response and a single linear term for the log-transformed exposure, with a lag period of 36 years, i.e., log{*E*(PCD_*t*_)} = 4.899 + 0.328log(AI_*t*–36_), which yielded a minimum AIC of 278.8 and a coefficient of determination of 0.94. Reverting to the original scale, the asbestos-based prediction model resulted in the power function *E*(PCD_*t*_) = 134.3AI_*t*–36_^0.33^. This model was used to predict the expected annual number of pleural cancer deaths among men across the upcoming period 2012–2038.

**Figure 3:**
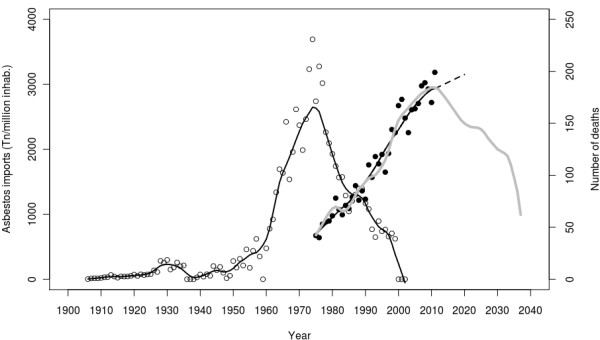
**Annual raw per capita asbestos imports recorded in Spain from 1906 to 2002 (black circles), together with annual pleural cancer deaths in men (black points) from 1975 to 2011 and the APC predicted for 2011–2020.** Grey line: deaths prediction based on imports. Black line (left): lowess smoothing of imports. Black line (rigth): lowess smoothing of observed (continuous) and predicted (dashed) pleural cancer deaths.

Shown in Figure 3 are the annual per capita asbestos imports recorded in Spain for the period 1906–2002 (black circles), along with the pertinent lowess smoothing (black line). The right part of the figure shows the annual number of pleural cancer deaths in men observed from 1975 to 2011 (black points), with a black line showing the lowess smoothing of observed (continuous) and predicted (dashed) pleural cancer deaths. Future pleural cancer deaths among men, as estimated by the log model based on asbestos imports, were 145 to 160 deaths/year for the periods 2011–2015 and 2016–2020 respectively. These figures were lower than those obtained with the APC models (187 and 193 deaths). Estimates by five-year period have been added to Table 1 to facilitate comparison.

## Discussion

Pleural cancer deaths increased across the study period, rising from 491 in 1976–1980 to 1,249 in 2006–2010. Around 1,319 pleural cancer deaths (264 deaths/year) are predicted for the five-year period 2016–2020. Forecasts up to 2020 indicate that this increase will continue, though the age-adjusted rates already show a trend towards a levelling-off in male mortality from 2001–2005, a trend corresponding to the lower risk in post-1960 generations. The lower rates and different mortality trend registered by women would appear to indicate that occupational exposure is possibly the single factor having most influence on this trend.

Previously published predictions [17] estimated that there would be 636 male pleural cancer deaths (95% CI 499–656) in the period 2007–2011 and a further 685 (95% CI 497–960) in the period 2012–2016. Current data show that in the five-year period 2006–2010 there were 897 pleural cancer deaths in men, which would correspond to 642 deaths due to pleural mesothelioma, assuming a mesothelioma/pleural cancer coefficient of 0.73. In the five-year period 2006–2010, there were approximately 261 more deaths than the figure predicted in 2008 (underestimate of 41%).

Unlike other studies [9,15,26], the APC computation of the prediction did not consider asbestos imports or their decline from the 1980s until 2002 (the year in which they came to a halt). The prediction did, however, consider the decreasing mortality in post-1950 cohorts, a decrease visible in the age-specific mortality rates and cohort effect shown in Figure 2. The lag between asbestos imports and pleural cancer deaths shown in Figure 3 (taking the maximum of both series into account) and confirmed by the modelling strategy, lasted 36 years, which corresponds to the induction period for mesothelioma [27]. Future male pleural cancer mortality estimated by the asbestos import-based model accurately predicted the increase in pleural cancer deaths until 2008 but predicted a decrease in deaths from 2010 onwards.

The number of pleural cancer deaths among men predicted for the period 2011–2020 is 1898 under the APC-based model versus 1680 under the asbestos import-based model. The import-based model underestimates the deaths observed during 2011. This model lends excessive weight to the effect of the decline in asbestos imports, as a consequence of the simple statistical relationship established.

Because of the rarity of primary malignant tumours of the pleura other than mesothelioma, the number of deaths coded as pleural cancer could be considered a rough estimate of mesothelioma cases [28]. Mesothelioma mortality estimates and projections are based on the ratio of pleural mesothelioma mortality to pleural cancer mortality. Thus, a factor of 0.81 was used in France to estimate pleural mesothelioma cases from the total number of pleural cancer deaths [15], and a factor of 0.73 was used in Italy [9].

From 2006 to 2011, there were 1096 male deaths in Spain coded as cancer of the pleura, and of these, 849 (77%) were coded as pleural mesotheliomas. In women, this percentage was 63% (436 pleural cancer deaths, 273 mesotheliomas). If these factors are applied to the pleural cancer deaths predicted for the period 2016–2020, then a total of 965 mesothelioma cases (742 in men and 223 in women) is obtained (193 deaths/year).

The number of mesothelioma deaths predicted for Spain is lower than that for other countries in the region [9,15,16]. This is because this country’s asbestos consumption has historically been lower. The use of bulk asbestos in Spain started in the early 1960s, at a later date than the rest of Europe. Consumption witnessed a remarkable increase until 1974, sharing highs in those years with other European countries. Thenceforth, however, following the same trend as seen in other countries, consumption went into a steady decline that continued until the product’s ban in 2002, with a slight increase at the end of the 1980s. Overall consumption in Spain was one-third lower than that of France and Italy, and half that of the United Kingdom [7,8].

Spanish records however show far fewer mesothelioma-related deaths than would have been expected, i.e., 74% fewer than in France, 79% fewer than in Italy, and 88% fewer than in the United Kingdom in the year 2000. Several hypotheses can be postulated to explain this difference. Attention to the risks arising from exposure to asbestos in Spain was almost non-existent until the 1980s, making it difficult for this diagnosis to be assigned as the cause of death, in contrast to other countries in the region [29]. On the other hand, each country’s prevailing economic risk activities are decisive, conditioning differences in asbestos exposure [30,31].

Part of the increase in cases in Spain in the 1990s could be due to improvements in diagnosis of mesothelioma as a cause of death. It seems more plausible, however, that this increase is attributable to the effects of increased asbestos consumption, and that the introduction of regulatory measures and prohibitions were delayed in Spain and that this in turn led to the effects of exposure being extended.

The first preventive measures targeting asbestos use in Spain were implemented in 1984 [32] and the ban on the use, production and marketing of asbestos fibres and any products containing these was introduced in 2001 [33] and came into effect in December 2002. Subsequently, minimum health and safety requirements were brought in 2006 to cover work entailing a risk of exposure to asbestos [34]. Such work essentially comprises the removal of installed asbestos, the maintenance, rehabilitation and demolition of buildings with asbestos-containing materials, and the transport, treatment and destruction of asbestos-containing waste.

Changes in death-coding criteria can also affect mortality trends. The decline in mortality rates in the five-year period 1986–1990 and the observed period effect (Figure 2d) are probably related to coding changes that took place in Spain at the time (when death-coding duties were transferred to the Autonomous Regions).

Møller’s proposal for estimating future mortality was used in this study [22]. This method, based on the classic “generalised linear model” [25], is implemented in the Norpred package, which has not only been successfully used in several European countries [35-37] but has also been empirically shown to improve prediction validity [22]. Comparative prediction-quality studies using different methods have recommended the use of Norpred in cases such as ours, where there is a cohort effect identified by age-period-cohort analysis [38].

The disappearance of occupational exposure to asbestos should lead to a reduction in the number of deaths among men to levels equivalent to those recorded for women, which are two thirds lower. Mortality caused by community exposure to a carcinogenic risk factor such as asbestos is characterised by a great degree of inertia, and its catastrophic consequences will persist and remain in evidence until the disappearance of the last surviving representatives of the exposed cohorts. To this must be added the fact that there were no preventive measures aimed at reducing worker exposure, nor any emission controls until as late as 1984. It can thus be assumed that occupationally-related deaths due to pleural mesothelioma will continue to occur in Spain until at least 2040, bearing in mind the fact that in 2006 the life expectancy of Spanish men born in the 1960s was 37.4 years. Moreover, account must be taken of the enormous amount of asbestos which was used in the construction of buildings (mostly offices, entertainment venues, public facilities and car parks) from 1965 to 1985 [23] and which will be mobilised for subsequent demolition or maintenance purposes. Improved education regarding protection from exposure during the demolition or disposal of asbestos-containing material as well as more adequate measures for reducing risk of mesothelioma are thus called for [26]. Similarly, the qualification and training of health care workers in disease detection and diagnosis must also be improved so as to ensure that these diseases do not go undetected.

## Conclusions

Predictions for the five-year period 2016–2020 indicate a total of 1319 pleural cancer deaths (264 deaths/year). Forecasts up to 2020 indicate that deaths will continue to rise, though the age-adjusted rates show a levelling-off in male mortality from 2001–2005, corresponding to the lower risk in post-1960 generations. The different mortality trend registered by women would appear to indicate that occupational exposure is possibly the single factor having most influence on this trend. The catastrophic consequences of human exposure to asbestos are set to persist and remain in evidence until the last surviving members of the exposed cohorts have disappeared. It can thus be assumed that occupationally-related deaths due to pleural mesothelioma will continue to occur in Spain until at least 2040.

## Competing interests

The authors declare that they have no competing interests.

## Authors’ contributions

GLA and MGG, were involved in designing the study. GLA, RPB and JGP performed the statistical analysis. GLA wrote the first draft of the manuscript, to which all authors subsequently contributed. AMN, RR, MC, EF, MJM made contribution to statistical analyses and interpretation of results, and revised the manuscript for important intellectual content. All authors read and approved the final manuscript.

## Pre-publication history

The pre-publication history for this paper can be accessed here:

http://www.biomedcentral.com/1471-2407/13/528/prepub
